# Impact of bacterial co-isolation on treatment initiation and long-term prognosis of patients with nontuberculous mycobacterial pulmonary disease: analysis of a prospective cohort study

**DOI:** 10.1186/s12879-024-10006-x

**Published:** 2024-10-08

**Authors:** Joong-Yub Kim, Sung A Kim, Kwonhyung Hyung, Jae-Joon Yim, Nakwon Kwak

**Affiliations:** 1 Division of Pulmonary and Critical Care Medicine, Department of Internal Medicine, Seoul National University Hospital, Seoul, Republic of Korea; 2 Department of Internal Medicine, Seoul National University College of Medicine, Seoul, Republic of Korea

**Keywords:** Nontuberculous mycobacterial pulmonary disease, Bacterial co-isolation, Treatment initiation, *Pseudomonas*, Potentially pathogenic microorganisms

## Abstract

**Background:**

Nontuberculous mycobacterial pulmonary disease (NTM-PD), a chronic respiratory condition, presents a growing challenge globally. Uncertainties exist regarding the impact of concurrent bacterial co-isolation on treatment initiation and long-term prognosis.

**Methods:**

This study analysed data from participants enrolled in an ongoing prospective observational cohort study on NTM-PD (NCT01616745) between 1 July 2011, and 31 December 2022, who provided sputum samples for bacterial culture at enrolment. Identification of potential pathogenic microorganisms (PPMs) was defined as a positive bacterial culture. Clinical characteristics were compared between NTM-PD patients with *Pseudomonas*, non-pseudomonal PPMs, and those without PPM co-isolation. Cox proportional hazard regression models were employed to assess the association of bacterial co-isolation with rates of NTM-PD treatment initiation and all-cause mortality.

**Results:**

Overall, 453 patients (median age, 62 years; 30% male) were included in the analysis. PPMs were co-isolated in 77 patients (17%), including 13 with *Pseudomonas* species. Co-isolation of *Pseudomonas* was associated with a significantly higher erythrocyte sedimentation rate (*P* = 0.02) and St. George’s Respiratory Questionnaire score (*P* = 0.01). Non-pseudomonal PPM co-isolation was significantly associated with a higher likelihood of NTM-PD treatment initiation (adjusted hazards ratio [aHR], 1.56, 95% confidence interval [CI], 1.03–2.36, *P* = 0.036), whereas co-isolation of *Pseudomonas* was independently correlated with increased all-cause mortality (aHR, 3.25, 95% CI, 1.08–9.84, *P* = 0.037).

**Conclusions:**

Our findings emphasize the importance of microbial surveillance, as bacterial co-isolation affects treatment initiation and prognosis in patients with NTM-PD.

## Background

Nontuberculous mycobacterial pulmonary disease (NTM-PD) is a chronic respiratory condition caused by mycobacterial species other than the *Mycobacterium tuberculosis* complex and *M. leprae* [1]. NTM-PD frequently manifests in patients with prior tuberculosis, chronic obstructive pulmonary disease, and bronchiectasis. However, it can also emerge spontaneously in patients lacking discernible underlying conditions due to the ubiquitous presence of NTM in the environment [2, 3].

Bacterial colonization, particularly by *Pseudomonas aeruginosa*, significantly heightens the risk of acute exacerbation and mortality in non-cystic fibrosis bronchiectasis [4]. Patients harbouring *P. aeruginosa* exhibit higher computed tomography (CT) severity scores, experience more frequent exacerbations, and demonstrate lower mean forced expiratory volume in 1 s than do patients with no identified pathogens. Conversely, patients with bacterial colonization other than *Pseudomonas* fall between these two groups [5]. Consequently, prognostic scores, such as the bronchiectasis severity index, incorporate the colonization status of *Pseudomonas* and other organisms into their scoring systems to stratify the risk of mortality and hospitalization of patients [6].

NTM-PD is closely associated with non-cystic fibrosis bronchiectasis. The nodular bronchiectatic form is the most common radiographic pattern in NTM-PD, and a significant portion of newly diagnosed NTM-PD patients may also have underlying bronchiectasis [7, 8]. Consequently, bacterial co-isolation is prevalent in NTM-PD, in up to 45% cases, often involving *Staphylococcus aureus*, *P. aeruginosa*, and *Haemophilus influenzae* [9, 10]. However, the impact of bacterial co-isolation in patients with NTM-PD remains unclear. A retrospective cohort study suggests NTM-PD patients with bacterial co-isolation are less likely to initiate treatment for NTM-PD but are more susceptible to bronchiectasis exacerbation [9]. These seemingly conflicting observations prompt questions about the influence of bacterial co-isolation on the long-term prognosis of patients with NTM-PD, and whether such effects vary depending on the bacterial species involved. Thus, we aimed to address these queries by analysing an ongoing prospective observational cohort of patients with NTM-PD.

## Methods

### Study population

The study cohort comprised participants enrolled in an ongoing prospective observational cohort study (NCT01616745) initiated on July 1, 2011, at Seoul National University Hospital, a tertiary referral university-affiliated hospital in South Korea. All enrolled patients provided written informed consent. Patients aged 20 to 80 years, satisfying the diagnostic criteria of NTM-PD as outlined in 2007 American Thoracic Society/Infectious Disease Society of America guidelines, were enrolled in this cohort [1]. Detailed inclusion criteria are available elsewhere [11]. Clinical, radiological, and laboratory data were obtained upon study enrolment. The current analysis included participants enrolled between 1 July, 2011, and 31 December, 2022. Exclusion criteria encompassed individuals who did not provide sputum samples for bacterial culture at enrolment, those with a follow-up duration < 6 months, and those who immediately underwent treatment for NTM-PD upon enrolment. This analysis received approval from the Institutional Review Board of Seoul National University Hospital (No: H2308-072-1457). Some patients from previous studies were also included in this analysis [7, 12–20].

## Study variables and outcomes

We collected demographic, microbiological, radiological, and clinical data, including age, sex, body mass index (BMI), causative NTM species, acid-fast bacilli (AFB) smear positivity, sputum bacterial culture results, presence of cavities on chest CT scan, medical history, comorbidities, St. George’s Respiratory Questionnaire (SGRQ) score, a patient-reported health-related quality of life questionnaire widely used in pulmonary conditions [21, 22], laboratory test results, and pulmonary function at study enrolment. Positive bacterial culture was defined as the identification of potential pathogenic microorganisms (PPMs), which included bacteria frequently causing respiratory infections such as gram negative rods [23]. Positive bacterial cultures were subsequently categorized as *Pseudomonas* and non-pseudomonal co-isolation. We also reviewed antibiotic prescription history to identify the start date of treatment for NTM-PD. The initiation of treatment or the clinician’s intention to treat was defined as NTM-PD progression. Once antibiotic treatment was administered, the regimen composition was determined based on the international guidelines [1, 24]. Patients were censored at death or on 31 May, 2023, whichever occurred first. Vital status and date of death were collected from a database verified by the Ministry of the Interior and Safety of South Korea.

### Statistical analysis

Descriptive statistics were employed to compare clinical characteristics among patients with *Pseudomonas* co-isolation, non-pseudomonal PPM co-isolation, and those without PPM co-isolation. Categorical variables were presented as frequencies with proportions, and continuous variables as medians with the first and third quartiles (Q1–Q3). Differences between groups were compared using the Kruskal–Wallis test, Pearson’s chi-square test, or Fisher’s exact test, as indicated. Cox proportional hazards regression models were used to determine whether bacterial co-isolation was associated with the rates of NTM-PD treatment initiation and mortality. For multivariable analyses, factors associated with treatment initiation and mortality, such as *Pseudomonas* co-isolation as identified in the literature, were adjusted [16, 20, 25, 26]. *P*-values < 0.05 for two-sided tests were considered statistically significant. All statistical analyses were performed using R software version 4.3.3 (R Foundation for Statistical Computing, Vienna, Austria).

## Results

### Baseline characteristics

During the study period, 522 patients were enrolled. After excluding 24 patients who did not provide sputum for bacterial culture, 22 patients with a follow-up duration < 6 months, and 23 who initiated treatment for NTM-PD at enrolment, 453 patients were finally included in the analysis (Table 1). The median age was 62 (Q1–Q3, 56–68) years, with men comprising 29.8% of the study population. Detailed clinical characteristics at enrolment are summarized in Table 1.

**Table 1:** Clinical characteristics stratified by bacterial co-isolation

Characteristics	Total (*N* = 453)	No growth (*n* = 376)	Pseudomonas (*n* = 13)	non-Pseudomonal PPMs (*n* = 64)	*P*
Age, years	62 (56–68)	61 (55–68)	63 (56–66)	64 (60–71)	0.099
Men	135 (29.8)	107 (28.5)	2 (15.4)	26 (40.6)	0.074
BMI, kg/m²	20.9 (19.1–22.4)	21.0 (19.2–22.5)	20.1 (19.0-22.2)	20.4 (18.8–21.9)	0.287
ESR, mm/hr (*n* = 450)	20.0 (12.0–32.0)	19.0 (11.0–31.0)	40.0 (19.0–44.0)	21.5 (15.0-27.5)	0.018
AFB smear positivity	83 (18.3)	69 (18.4)	2 (15.4)	12 (18.8)	0.959
Mycobacterial species					
*M. avium*	152 (33.6)	124 (33.0)	4 (30.8)	24 (37.5)	0.760
*M. intracellulare*	127 (28.0)	105 (27.9)	2 (15.4)	20 (31.3)	0.506
*M. abscessus subsp. abscessus*	37 (8.2)	33 (8.8)	2 (15.4)	2 (3.1)	0.196
*M. abscessus subsp. massiliense*	18 (4.0)	14 (3.7)	0 (0.0)	4 (6.3)	0.480
*M. kansasii*	9 (2.0)	8 (2.1)	0 (0.0)	1 (1.6)	0.835
Radiographic phenotype					
Fibrocavitary	64 (14.1)	58 (15.4)	1 (7.7)	5 (7.8)	0.215
Nodular bronchiectatic	381 (84.1)	312 (83.0)	12 (92.3)	57 (89.1)	0.335
Presence of cavity	105 (23.2)	93 (24.7)	1 (7.7)	11 (17.2)	0.169
BACES score (*n* = 450)	2 (1–2)	1 (1–2)	2 (1–2)	2 (1–3)	0.160
Smoking history					0.450
Current	10 (2.2)	8 (2.1)	0 (0.0)	2 (3.1)	
Past	92 (20.3)	74 (19.7)	1 (7.7)	17 (26.6)	
Never	351 (77.5)	294 (78.2)	12 (92.3)	45 (70.3)	
Prior tuberculosis (*n* = 441)	151 (34.2)	133/366 (36.3)	4/13 (30.8)	14/62 (22.6)	0.104
Prior pertussis (*n* = 316)	17 (5.4)	10/271 (3.7)	2/7 (28.6)	5/38 (13.2)	0.001
Prior measles (*n* = 340)	76 (22.4)	64/290 (22.1)	2/8 (25.0)	10/42 (23.8)	0.953
Comorbidities					
COPD	22 (4.9)	18 (4.8)	0 (0.0)	4 (6.3)	0.626
Diabetes (*n* = 450)	27 (6.0)	22/373 (5.9)	1/13 (7.7)	4/64 (6.3)	0.961
GERD (*n* = 438)	36 (8.2)	27/365 (7.4)	1/11 (9.1)	8/62 (12.9)	0.343
SGRQ score	14.5 (6.8–25.9)	14.1 (6.6–25.1)	35.1 (16.1–42.0)	17.9 (7.2–29.3)	0.009
FEV1% predicted (*n* = 434)	100 (88–112)	100 (88–112)	98 (77–117)	105 (93–117)	0.236
FVC% predicted (*n* = 434)	95 (85–105)	95 (85–104)	100 (77–118)	94 (84–106)	0.756

Data presented in medians with first and third quartiles or numbers with proportions. Unless otherwise noted, the denominator of each variable corresponds to the number of patients in the specific group being analyzed

*Abbreviations* PPMs, potential pathogenic microorganisms; BMI, body mass index; ESR, erythrocyte sedimentation rate; AFB, acid-fast bacilli; BACES, BMI, Age, Cavity, ESR, Sex; COPD, chronic obstructive pulmonary disease; GERD, gastroesophageal reflux disease, FEV1, forced expiratory volume in 1 s; FVC, forced vital capacity

Overall, PPMs were isolated from 77 (17%) patients (Table 2). Among these, 13 (17%) patients had *Pseudomonas* species, whereas 64 (83%) had PPMs other than *Pseudomonas*. Patients with *Pseudomonas* species exhibited significantly higher erythrocyte sedimentation rate (ESR) and SGRQ scores than those with non-pseudomonal PPMs (*P* = 0.01 for ESR; *P* = 0.02 for SGRQ scores) or no bacterial co-isolation (*P* = 0.008 for ESR; *P* = 0.003 for SGRQ scores). Additionally, patients with *Pseudomonas* had a significantly higher proportion of pertussis history, compared with those without *Pseudomonas* co-isolation (28.6% vs. 4.9%, *P* = 0.049).

**Table 2:** Identified bacterial species from sputum at baseline

Species	*N* = 77 (%)
*Klebsiella* species	41 (53.2)
*pneumoniae* ss. *pneumoniae*	36 (46.8)
*pneumoniae* ss. *ozaenae*	1 (1.3)
*aerogenes*	2 (2.6)
*oxytoca*	2 (2.6)
*Pseudomonas* species	13 (16.9)
*aeruginosa*	8 (10.4)
*fluorescens*	4 (5.2)
*putida*	1 (1.3)
*Staphylococcus aureus*	8 (10.4)
*Streptococcus* species	7 (9.1)
*viridans* group	3 (3.9)
*pneumoniae*	2 (2.6)
*salivarius* ss. *salivarius*	1 (1.3)
*parasanguinis*	1 (1.3)
*Enterobacter cloacae*	2 (2.6)
*Haemophilus influenzae*	1 (1.3)
Others	5 (6.5)

### Bacterial co-isolation and the rate of NTM-PD treatment initiation

During a median follow-up of 5.4 (Q1–Q3, 2.5–8.0) years, 180 patients initiated treatment for NTM-PD (Table 3). The median time to NTM-PD treatment initiation was 2.8 (Q1–Q3, 1.0–6.0) years. When previously reported risk factors of treatment initiation such as female sex, ESR elevation (> 15 mm/h in men and 20 mm/h in women), percentage of forced expiratory volume in 1 s (FEV1%), presence of cavity, and AFB smear positivity were adjusted, co-isolation of non-pseudomonal PPMs was significantly associated with a higher likelihood of initiating NTM-PD treatment (adjusted hazard ratio [aHR], 1.56, 95% confidence interval [CI], 1.03–2.36, *P* = 0.036), whereas *Pseudomonas* co-isolation was not (aHR, 0.98, 95% CI, 0.43–2.24, *P* = 0.96) (Table 4).

**Table 3:** Outcomes by bacteria type identified from baseline sputum

Characteristics	Total (*N* = 453)	No growth (*n* = 376)	Pseudomonas (*n* = 13)	non-Pseudomonal PPMs (*n* = 64)	*P*
Median follow-up duration (Q1–Q3)	5.4 (2.5–8.0)	5.3 (2.5–7.9)	7.7 (3.5–11.0)	5.5 (2.9–8.3)	0.183
NTM-PD progression	180 (39.7)	145 (38.6)	6 (46.2)	29 (45.3)	0.530
Death	48 (10.6)	36 (9.6)	4 (30.8)	8 (12.5)	0.044

*Abbreviations* PPM, potential pathogenic microorganism; NTM-PD, nontuberculous mycobacterial pulmonary disease

**Table 4:** Impact of bacterial co-isolation on NTM-PD treatment initiation

Outcomes	NTM-PD treatment initiation			
	HR (95% CI)	*P*	adjusted HR (95% CI)	*P*
non-pseudomonal PPM co-isolation	1.20 (0.81–1.79)	0.360	1.56 (1.03–2.36)	0.036
*Pseudomonas* co-isolation	1.03 (0.46–2.33)	0.941	0.98 (0.43–2.24)	0.96
Presence of cavity	2.79 (2.06–3.77)	< 0.001	2.70 (1.95–3.75)	< 0.001
Female sex	1.32 (0.95–1.83)	0.104	1.76 (1.23–2.52)	0.002
ESR elevation	1.95 (1.44–2.64)	< 0.001	1.67 (1.22–2.30)	0.002
AFB smear positivity	1.48 (1.05–2.09)	0.025	1.31 (0.91–1.89)	0.15
FEV1%	0.98 (0.98–0.99)	< 0.001	0.99 (0.98–0.99)	< 0.001

*Abbreviations* HR, hazard ratio; CI, confidence interval; PPM, potential pathogenic microorganism; ESR, erythrocyte sedimentation rate; AFB, acid-fast bacilli; FEV1%, forced expiratory volume in 1 s

## Bacterial co-isolation and all-cause mortality

During a median survival of 6.5 (Q1–Q3, 3.3–9.0) years, 48 patients died (Table 3). When factors associated with mortality including BMI < 18.5 kg/m^2^, age ≥ 65 years, presence of cavity, ESR elevation, FEV1%, and sex were adjusted, the co-isolation of *Pseudomonas* was independently associated with increased all-cause mortality (aHR, 3.25, 95% CI, 1.08–9.84, *P* = 0.037) whereas non-pseudomonal PPM co-isolation was not (aHR, 0.94, 95% CI, 0.42–2.09, *P* = 0.88) (Table 5).

**Table 5:** Impact of bacterial co-isolation on all-cause mortality in patients with NTM-PD

Outcomes	All-cause mortality			
	HR (95% CI)	*P*	adjusted HR (95% CI)	*P*
non-pseudomonal PPM co-isolation	1.15 (0.53–2.48)	0.720	0.94 (0.42–2.09)	0.88
*Pseudomonas* co-isolation	2.26 (0.80–6.37)	0.122	3.25 (1.08–9.84)	0.037
Presence of cavity	2.49 (1.40–4.41)	0.002	1.63 (0.87–3.07)	0.13
Female sex	0.22 (0.12–0.41)	< 0.001	0.30 (0.16–0.60)	< 0.001
ESR elevation	5.34 (2.39–11.95)	< 0.001	3.24 (1.40–7.49)	0.006
Age ≥ 65 years	3.91 (2.03–7.51)	< 0.001	2.89 (1.43–5.83)	0.003
BMI < 18.5 kg/m^2^	1.93 (1.02–3.65)	0.043	1.27 (0.63–2.58)	0.51
FEV1%	0.97 (0.96–0.98)	< 0.001	0.98 (0.97–0.998)	0.026

*Abbreviations* HR, hazard ratio; CI, confidence interval; PPM, potential pathogenic microorganism; ESR, erythrocyte sedimentation rate; BMI, body mass index; FEV1%, forced expiratory volume in 1 s

## Discussion

In this analysis of a prospective observational cohort study, we examined 453 patients with NTM-PD to evaluate the impact of bacterial co-isolation on NTM-PD treatment initiation and long-term prognosis. Overall, PPMs were identified from 17% of patients at study enrolment. *Pseudomonas* co-isolation was associated with significantly elevated levels of inflammatory markers, such as ESR, and more severe symptoms, compared with patients without *Pseudomonas* co-isolation. Non-pseudomonal PPM co-isolation was linked to higher rates of NTM-PD treatment initiation, whereas patients with *Pseudomonas* co-isolation were four times more susceptible to mortality.

*Pseudomonas* has been associated with a higher inflammatory response and poor long-term prognosis in patients with non-cystic fibrosis bronchiectasis. Previous studies have demonstrated that viable *P. aeruginosa* enhances the expression of pro-inflammatory cytokines such as interleukin-6 and tumour necrosis factor-α in the host [27, 28]. Moreover, this inflammatory response induced by bacteria has been identified as the major component of the ‘vicious vortex’ of bronchiectasis, leading to airway dysfunction and lung destruction [29]. Multiple clinical cohort studies have reaffirmed the association between the presence of *Pseudomonas* and greater pulmonary function decline, more frequent acute exacerbations, and increased hospitalizations [4, 5, 30]. Considering that the majority of patients in this study exhibited the nodular bronchiectatic phenotype, our findings align those of previous studies and underscore the significance of *Pseudomonas* in terms of mortality in NTM-PD.

In contrast to the effect of *Pseudomonas* on mortality, the co-isolation of *Pseudomonas* did not affect NTM-PD treatment initiation, whereas the co-isolation of non-pseudomonal PPM was associated with early initiation of treatment. These findings contradict those of a Japanese study that demonstrated that co-infections with *Pseudomonas* or non-pseudomonal PPM were associated with a slower initiation of treatment for NTM-PD [9]. Our findings can be explained as follows: firstly, PPMs have been shown to induce an immune response in the host, including uncoordinated T-cell activation and excessive cytokine release, resulting in lung tissue damage and potential exacerbations [31]. This immune response and subsequent tissue damage could create an opportunistic environment for NTM proliferation, thereby advancing the progression of NTM-PD. Secondly, clinicians may have inadvertently initiated NTM-PD treatment because of worsening symptoms and radiographic findings caused by PPMs. Further research is needed to confirm these hypotheses.

Conversely, the lack of an influence of *Pseudomonas* on NTM-PD treatment initiation, in contrast to non-pseudomonal PPMs, suggests different actions of *Pseudomonas* in NTM-PD. The baseline characteristics and disease severity of patients with *Pseudomonas* were comparable to those of patients without *Pseudomonas*, except for a prior history of pertussis. Hence, the presence of *Pseudomonas* did not necessarily indicate less severe NTM-PD compared to those with non-pseudomonal PPMs. Although it is challenging to comprehend this issue, insights can be gained from in vitro studies. Biofilm development assays demonstrated that the addition of *P. aeruginosa* is associated with a significant reduction in *M. abscessus* biofilm development [32]. Moreover, when grown in biofilms of both species, a notable inhibition of *M. abscessus* abundance was observed, whereas *P. aeruginosa* levels remained unchanged [33]. This antagonistic interplay between the two species appears to favour the proliferation of *P. aeruginosa.* Considering the formidable challenge of eradicating *Pseudomonas* [34, 35], it is plausible that its presence could have suppressed NTM and impeded the progression of NTM-PD, although further research is required to reveal the complex interactions between NTM and *Pseudomonas* within the host.

Our findings underscore the importance of performing bacterial culture and incorporating its results when managing patients with NTM-PD. Although the proportion of patients with PPM co-isolation in our study was smaller than that in other studies [9], a significant number still demonstrated such co-isolation. Given the adverse prognostic implications associated with *Pseudomonas*, and the link between non-pseudomonal co-isolation and NTM-PD progression, monitoring of bacterial profiles may be helpful. Early identification and targeted antibiotic therapy for patients with PPM co-isolation could offer potential benefits in mitigating NTM-PD progression and enhancing overall prognosis.

The main strength of this study lies in the analysis of a prospectively enrolled cohort of patients with NTM-PD. However, our findings are limited by the exclusion of patients who failed to provide sputum samples at enrolment. Moreover, the longitudinal changes of bacterial co-isolation were not assessed. One study reported an annual acquisition rate of *P. aeruginosa* of 3.3% [8]. Additionally, the number of patients with PPM co-isolation was very small, with even fewer exhibiting co-isolation of *Pseudomonas.* Several factors may have contributed to this, including the cohort’s focus on patients with NTM-PD rather than those with bronchiectasis, the relatively low disease severity as indicated by the BACES score, a low proportion of patients with chronic obstructive pulmonary disease, and the use of sputum cultures instead of more sensitive respiratory specimens, such as bronchoalveolar lavage fluid. Consequently, assessment of the specific effects of individual species was not possible due to the small number of patients with *Pseudomonas* co-isolation. For instance, although *P. putida* and *P. fluorescens* are known pathogenic species that can cause pneumonia and other infections [36, 37], their impact on the host may differ from that of *P. aeruginosa*. However, despite the small number of patients with *Pseudomonas* co-isolation at diagnosis, this study clearly underscores its significant association with poor long-term prognosis. Due to limited understanding regarding the link between a history of pertussis and subsequent *Pseudomonas* colonization, we were unable to fully explain why patients with *Pseudomonas* co-isolation had a significantly higher proportion of pertussis history, despite only two such cases. Further research is needed to clarify the underlying mechanisms. Furthermore, we only analysed conventional bacterial cultures. Future studies that integrate longitudinal and microbiome data may provide a more holistic view of the impact of bacterial co-isolation on NTM-PD.

## Conclusions

In patients with NTM-PD, *Pseudomonas* co-isolation is linked to hyperinflammation, worsening symptoms, and increased mortality, whereas co-isolation of non-pseudomonal PPMs is associated with a higher likelihood of NTM-PD treatment initiation.

## Data Availability

The datasets used are available from the corresponding author on reasonable request after IRB approval.

## Abbreviations

aHR: Adjusted hazard ratio
AFB: Acid-fast bacilli
BMI: Body mass index
CI: Confidence interval
CT: Computed tomography
ESR: Erythrocyte sedimentation rate
NTM-PD: Nontuberculous mycobacterial pulmonary disease
PPMs: Potential pathogenic microorganisms
SGRQ: St. George’s Respiratory Questionnaire
